# Nest Depth and Height Are Associated with Breeding Outcomes in the Small Bee-Eater (*Merops orientalis*): A Preliminary Field Study from Pakistan

**DOI:** 10.3390/ani16020186

**Published:** 2026-01-08

**Authors:** Asif Sadam, Muhammad Awais, Huijian Hu, Dongmei Yu, Yiming Hu

**Affiliations:** 1 Guangdong Key Laboratory of Animal Conservation and Resource Utilization, Institute of Zoology, Guangdong Academy of Sciences, Guangzhou 510260, China; saddamasif2@gmail.com (A.S.); yudongmei50@163.com (D.Y.); huyiming@giz.gd.cn (Y.H.); 2 Department of Zoology, Islamia College, Peshawar 25120, Pakistan; mawaisicp@gmail.com

**Keywords:** breeding success, clutch size, small bee-eater, cavity depth, nest height, Ballar stream

## Abstract

Nest architecture and surrounding habitat features can strongly influence the reproductive success of cavity-nesting birds; however, quantitative data from natural environments remain limited. We examined how nest structure and surrounding habitat features correlate with reproduction in the small bee-eater (*Merops orientalis*). A total of 38 natural nests were monitored during the breeding season. The Conway–Maxwell–Poisson model showed that cavity depth was a significant positive predictor of clutch size (β = 0.46 ± 0.22 SE, *p* = 0.036), whereas entrance diameter and nest height were not significantly related. Principal component analysis (PCA) of standardized cavity dimensions (cavity depth, entrance diameter, and nest height) showed that nest height (captured by PC2) was strongly associated with higher breeding success (OR = 0.002, *p* = 0.021), whereas overall cavity size (PC1) had a weaker, marginally positive correlation (OR = 3.87, *p* = 0.09). Habitat distance variables showed only weak, non-significant trends after accounting for multicollinearity. Nest structural traits explained more variation in reproductive performance than landscape variables (pseudo-R^2^ = 0.80 for clutch size; 0.59 for breeding success). Field monitoring of 38 nests showed a mean clutch size of 3.9 eggs, an overall hatching success of 77.5%, and a fledging success of 51.2%, yielding a 37.1% breeding success. Our results highlight the importance of conserving sandy streambanks and mitigating human disturbance in proximity to active nests to conserve breeding success in small bee-eaters. As these findings were based on one site and a single breeding season, broader generalizations will require replication across additional years and locations.

## Simple Summary

Small bee-eaters excavate nest cavities in open habitats, but little is known about the roles of cavity shape and surrounding habitat in their breeding ecology. In this study, we followed the fate of the eggs and chicks of the small bee-eater to determine whether cavity depth, entrance dimensions, nest height, and habitat in the vicinity (i.e., grasslands, farmlands, towns, or power lines) correlated with clutch size and reproductive success. We found that clutch size increased significantly with cavity depth, indicating that deeper cavities allowed females to lay more eggs. Breeding success was positively related to nest height, whereas entrance diameter and habitat distance to grasslands, farmlands, towns, and electric wires had little or no correlation. These results show that nest structural features, particularly cavity depth and height above ground, play a stronger role in breeding outcomes than surrounding landscape features. Therefore, conservation efforts should prioritize the preservation of suitable nesting habitats and minimize human disturbance around active breeding areas.

## 1. Introduction

Many animal species build specific structures to protect and rear their offspring during sensitive reproductive stages [1]. These nests differ greatly in construction, maintenance, form, size, composition, and location [2]. Even within the same species, nests may vary considerably in form and size, suggesting that behavioral differences and individual skills strongly influence nest structure. Such intraspecific variation has led to several hypotheses explaining how nest-site qualities, particularly in cavity-nesting species, can constrain nest size and, thereby, affect reproductive investment and performance [3,4]. The nest-building pattern is often indicative of the level of organization and precision displayed by a species in its construction. The relationship between construction effort, structural order, and physical limitations of the nesting site may dictate whether a nest effectively satisfies the functional demands of incubation and/or offspring brooding [1].

Many factors influence nest size; however, clutch size appears to be influenced by nest dimensions, with larger nests often accommodating larger clutches. Many studies have found a positive correlation between larger nests and clutch sizes [4,5,6]. This connection is especially captivating given that nests are usually built ahead of the onset of egg-laying, although the builders may still be working on the nest when the eggs are laid and even during the incubation period [7]. This poses a potential challenge for builders as they may not know the exact number of eggs laid during nest construction. One possible explanation is that both nest and clutch sizes are negatively correlated with laying date [8]. However, this does not fully account for the experimental evidence. Studies manipulating nest box size after egg-laying have demonstrated that clutch size can change in response to nest size, independent of confounding factors [4]. These findings suggest that larger nests may facilitate the production of larger clutches and support the rearing of more offspring. A female’s choice of nesting site can be directly connected to her clutch size. Females that build or choose larger nests may do so because they are preconditioned in a physiological sense to lay larger clutches [4]. Hence, the construction of a larger nest may be a response to a female’s decision to lay a larger clutch [6].

Choosing a nesting site is a vital part of how birds select their habitat [9]. Breeding habitat selection should be evaluated at both nest and foraging levels. For colonial species, such as small bee-eaters, two scales are especially important for excavation: (1) the micro-site scale (including streambank substrate, bank slope, and local vegetation), which limits burrow digging, affects nest microclimate, and influences predator access; and (2) the local foraging scale (such as nearby grasslands, farmland, and human-altered habitats), which determines food availability and foraging success [10,11].

Bee-eaters (Aves: Meropidae) include approximately 26 species with notable diversity in their social and breeding behaviors. Small bee-eaters (*Merops orientalis*) are widely distributed across Africa and southern Asia, from Egypt and the Arabian Peninsula through India and Sri Lanka, to Myanmar and southern China [12]. They inhabit open fields, sandy riverbanks, and lightly wooded areas that are suitable for nesting and foraging. Over 95% of its diet consists of flying insects such as beetles, dragonflies, butterflies, grasshoppers, and bugs [12,13,14]. Studies on the relationship between nest design and reproductive success have mainly focused on a select group of birds, particularly passerines that use nest boxes in controlled breeding experiments [4]. However, examining these relationships in natural settings offers a more realistic understanding of the behaviors related to nest construction and breeding success. This study was the first to quantitatively explore these relationships in small bee-eaters. In this study, we measured nest cavity characteristics (cavity depth, entrance diameter, and entrance height above the ground) and linear distances to key landscape features (grassland, farmland, towns, and electric infrastructure). Our goal was to determine whether (a) cavity dimensions restrict reproductive investment (clutch size) and (b) the landscape context around nests is correlated with breeding success through resource availability or disturbance. We tested two specific predictions: (1) deeper nest cavities may correlate with larger clutch sizes because of better microclimatic conditions or reduced risk from nest predators, and (2) nests located at higher elevations on the bankline are expected to have higher breeding success owing to lower ground attack rates from terrestrial predators and less disturbance from humans. Our goal was to understand how specific features of nest sites correlate with reproductive success in this species, shedding light on how to better conserve and manage cavity-nesting birds in complex landscapes.

## 2. Materials and Methods

### 2.1. Study Areas and Methods

**Figure 1:**
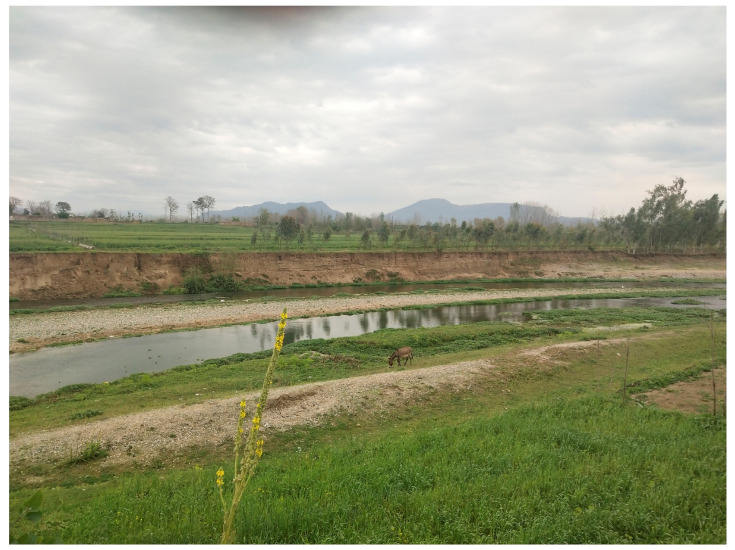
Photograph of the study area and the Ballar Stream.

We gathered data from the Ballar stream in the Mardan District of Khyber Pakhtunkhwa, Pakistan (Figure 1). Field data were collected during the 2024 breeding season. The stream is located 28 km northeast of Central Mardan (34°18′46.6488″ N; 72°9′1.1304″ E), and the region is irrigated. To the north is the Buner District, as well as an area protected by Malakand, and to the east is Sawabi, along with the Buner District. The Nowshera District is located to the south, the Charsadda District and the protected area of Malakand to the west [11]. The Ballar Stream is the principal watercourse in the region, flowing from the Sudhum Valley and joining the Kalpani River near Mardan. The area experiences a hot, semi-arid climate, with average summer temperatures of approximately 33 °C (May–June) and winter averages near 10 °C (December–January). Rainfall was highest during July–August and again in December–January. The Ballar Stream flows through a patchwork of cultivated and open grasslands interspersed with dwarf scrubs and small trees. Vegetated stream banks are typically dominated by fine sand and loam soil with low compaction, which have ideal burrow nesting properties in easily excavated substrates. There is little vegetation on the banks, and it consists mainly of grasses (*Cynodon dactylon* and *Saccharum spontaneum*), with bushes occurring only sporadically; they provide little cover, but there are some perching places available. Seasonal water-level fluctuations are marked, with the highest flows (July–August) immediately after the monsoon rains and reduced levels in March–May, when most of the nesting occurs. These seasonally induced phenomena influence the availability and stability of exposed sandy banks, which are exploited for breeding by small bee-eaters.

The study area was approximately 10 km long on the Ballar Stream, with both sides having appropriate nesting cliffs. We developed eight transects of ~1 km in length that were separated by approximately 200–300 m, following areas with access to the stream. Each transect was walked slowly at dawn (06:00–10:00) and late afternoon (16:00–18:00), the times of day when bee-eater activity peaks, thus increasing the detection probability. We searched the periphery of streams for excavation entrances and noted behavioral indicators (i.e., adults flying into or out of cavities). Each transect was checked three times during the breeding season to cover possible detection errors and reduce sampling bias. For each active nest, we measured (i) the nest-tunnel length (hereafter, cavity depth, the linear distance from the entrance rim to the egg chamber), (ii) entrance diameter (horizontal width of the opening), and (iii) nest height above the ground (vertical distance from the entrance to the stream bank). The internal chamber volume was not measured directly, and the “cavity size” was inferred from the external dimensions (cavity depth and entrance diameter). Gradient correction was not required because the cavities were nearly horizontal. These measurements were taken using a standard measuring tape and wooden ruler [12,13]. The depth of the cavity was measured after the clutch was completed, but as long as the cavities were still active, to avoid disturbing parturition. A bendable measuring tape was pushed down into the tunnel where the egg chamber was situated to measure the depth in a non-destructive manner. To provide direct access to the chambers, one side of the nest site was carefully dug out to expose the chamber without destroying the tunnel [12]. Eggs were measured in the nest, and the hole was closed again with a wooden plate so that it could be used later to record data on the nestlings. All nests were measured on calm, dry mornings within two days of clutch completion to avoid substrate breakage. Each measurement was repeated twice and averaged (±0.3 cm). The friable sand–loam substrate was stable during short visits, and repeated parental transit did not visibly alter entrance dimensions. Nests with evidence of collapse or damage were excluded from further consideration. Additionally, the distances between each nest and the nearest habitat structures (edge of grassland, edge of farmland, electric infrastructure, and nearest towns) were calculated in the field using a handheld laser rangefinder (±0.3 m accuracy for distances shorter than 1000 m) (Nikon Forestry Pro II, Nikon Corporation, Tokyo, Japan). The distances from the nest entrance to the nearest point of the habitat feature were recorded as linear (Euclidean) distances. Each measurement was performed twice, and the mean of these values was used for analysis to reduce errors.

### 2.2. Breeding Success

Nests with at least one egg were considered active (breeding). Nests with at least one fledging nestling were considered successful (successful nest). In the study area, known predators included the Indian gray mongoose (*Herpestes edwardsii*), various snakes (*Serpentes* spp.), house crow (*Corvus splendens*), jugger falcon (*Falco jugger*), and greater coucal (*Centropus sinensis*); however, predation was not observed. Thus, egg and nestling losses were ascribed to “predation without traces” (weather or other causes could also be involved).

These formulas were employed to determine the total egg volume, as well as hatching success, fledging success, and breeding success.

Volume = K v × LW^2^

In this case, “K v” is a constant = 0.51; “L,” as well as “W^2^” represent the width and length of the eggs, respectively.

$$Hatching\;success=\frac{Number\;of\;egg\;hatched}{Total\;egg\;laid}\times 100$$

$$Fledging\;success=\frac{Number\;of\;young\;fledged}{Total\;egg\;hatched}\times 100$$

$$Breeding\;success=\frac{Number\;of\;fledged\;young}{Total\;egg\;laid}\times 100$$

### 2.3. Statistical Analyses

#### 2.3.1. Correlation of Nest Structure with Clutch Size

Multicollinearity between predictors was checked based on variance inflation factors (VIFs) before fitting the models to prevent correlated variables from biasing model estimates. All predictive factors had acceptable VIF values (<3). The clutch size count data were analyzed using the Conway-Maxwell-Poisson (CMP) regression model, which allows for over- and underdispersion. Preliminary inspection of the variance-to-mean ratio revealed marked underdispersion, so that the CMP model was more appropriate than either the Poisson or negative binomial alternatives.

The response variable was clutch size per nest (*n* = 38); predictor variables included cavity depth (cm), entrance diameter (cm), and nest entrance height above ground (cm). Collinearity among predictors was evaluated using Pearson’s correlation and calculated VIF (VIF < 3 accepted).

The model coefficients were obtained using the maximum likelihood method with the COMPoissonReg package (R v4.3.2). Model fit was assessed using Akaike’s Information Criterion (AIC), adjusted McFadden pseudo-R^2^, and estimated dispersion parameter (ν).

To estimate generalization, repeated 5-fold cross-validation was performed (three repetitions and 15 folds in total). The CMP model was trained with 80% of the dataset and tested on the rest for each iteration. The predictive performance was estimated in terms of the root mean square error (RMSE), mean absolute error (MAE), and test-set log-likelihood. Non-converging folds (due to inadequate sample sizes) were removed from mean estimates. We tested the spatial clustering of nests along the Ballar Stream using Moran’s I on model residuals and found no evidence of significant spatial autocorrelation (*p* > 0.10). Analyses were conducted with one observation per nest to minimize temporal pseudo-replication over breeding stages.

#### 2.3.2. Correlation of Nest Structure and Breeding Success

For the comparability of coefficients and ease of interpretation, continuous predictors (depth, diameter, and height) were centered at zero and scaled to a standard deviation of one before analysis. Pairwise Pearson correlations and variance inflation factors (VIF) were conducted to test for multicollinearity across predictors. To be processed for dimension reduction, predictors with a high level of collinearity (VIF > 10 or r > 0.7) were selected. Given the collinearity between depth and diameter (r = 0.80, VIFs > 20), we conducted PCA on the standardized predictors. Factors with eigenvalues > 1, which explained ≥ 80% of the cumulative variance, were selected. The component loadings were used to elucidate the biological significance of each axis. PCA scores were added to the dataset as orthogonal predictors for further modeling. Logistic regression was used to analyze the binary cavity success results. Three models were compared: (1) Standard GLM using original predictors, (2) Firth penalized logistic regression to alleviate separation and small-sample bias, and (3) GLM based on PC1 and PC2 using them as covariates to correct for multicollinearity. Model fit was evaluated by Akaike’s Information Criterion (AIC) and McFadden’s pseudo-R^2^. Exponential coefficients were used for the Odds Ratios (ORs) and 95% confidence intervals (CIs). Effect directions were illustrated by predictions of success probabilities at levels across the range of PC1 and PC2 while holding the values of all other components at zero. Model diagnostics comprised tests for dispersion, overdispersion, and the influence of individual observations.

#### 2.3.3. Correlation of Habitat Features with Breeding Success

Because of the strong multicollinearity among habitat distance variables, we performed Principal Component Analysis (PCA) before modeling breeding success. The four continuous predictors (distance to grassland, farmland, towns, and electric wire) were standardized (mean 0, SD 1) using z-scores of the variables to remove scale effects. PCA was applied to the standardized matrix with correlation as the variable structure, and the number of retained components was based on eigenvalues (>1), scree plot examination, and cumulative variance extracted (>80%). The first three principal components (PCs) were selected, which explained 90.1% of the total variance in the data. Component loadings were explored to interpret the ecological significance of the PCs: PC1 was characterized by a gradient from proximity to urban environments to proximity to wires, PC2 contrasted the distance at farmland and grassland sites, and PC3 represented overall isolation away from both.

The PC scores representing uncorrelated axes were thereafter applied as predictors in a logistic regression with breeding success (1 = yes, 0 = no) as a binary response variable. We used a binomial distribution with a logit link to fit the models. We assessed model fit using residual deviance and compared models based on the Akaike Information Criterion (AIC) and significance of coefficients (Wald z-tests). Missing data were small (<5%) and were addressed through listwise deletion; sensitivity analyses using multiple imputation (m = 20) yielded similar results.

All analyses were performed using R statistical software (v4.3.2) with a significance level (α = 0.05). Data were visualized using the ggplot2 package (version 3.5.0) [14]. Results are presented as percentages and mean ± SD.

## 3. Results

### 3.1. Correlation of Nest Depth with Clutch Size

**Figure 2:**
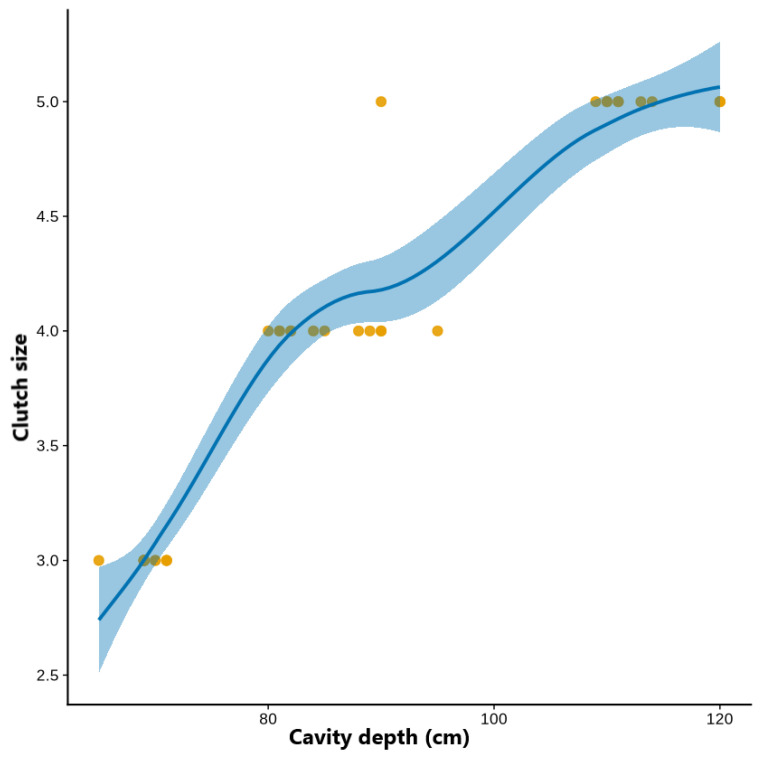
The association between clutch size and nest hole depth. The smooth line represents the fitted relationship between cavity depth and clutch size. The shaded band represents the 95% confidence interval (or confidence band) around the fitted relationship, showing uncertainty in the estimated mean clutch size across cavity depths.

The bee-eater nests had an average cavity depth of 86.7 ± 18.3 cm (range: 65–120 cm), entrance diameter of 10.9 ± 2.1 cm (range: 7–14 cm), and nest height of 201 ± 85.5 cm (range: 88–380 cm) across 38 nests (Table 1). In the CMP model, cavity depth was a significant positive predictor of clutch size (β = 0.457 ± 0.218 SE, z = 2.10, *p* = 0.036), representing an approximately 1.6-fold increase in the expected clutch size per centimeter of increase in depth. Entrance diameter had a weak, non-significant correlation in a positive direction (β = 2.50 ± 1.56 SE, *p* = 0.109), and nest height had no relationship (*p* = 0.659) (Table 2, Figure 2).

The model had a much better fit compared to an alternative Poisson model (AIC = 18.1 vs. 130.9), capturing the marked underdispersion in the data (ν = 76.5). The adjusted McFadden pseudo-R^2^ value was 0.804, indicating a strong explanatory power without overfitting.

Repeated 5-fold cross-validation (three repeats) confirmed a stable predictive performance (mean RMSE = 0.15 ± 0.16 eggs; MAE = 0.08 ± 0.11 eggs). Three folds (20%) did not converge because of small test sizes, but the results from the remaining folds were consistent, indicating that the model had a good generalization performance without overfitting. In general, clutch size increased with cavity depth, whereas the effects of entrance diameter or nest height were minimal.

### 3.2. Correlation of Nest Features with Breeding Success

**Figure 3:**
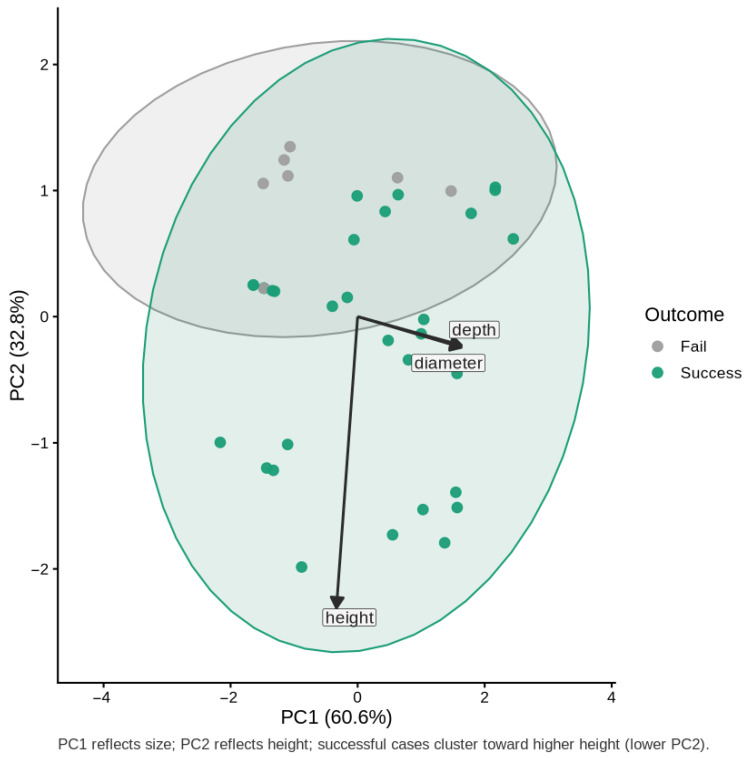
Principal component analysis (PCA) of standardized nest cavity traits and reproductive outcomes. The colored ellipses indicate 95% confidence regions for each outcome group, illustrating the multivariate dispersion and overlap in nest characteristics between successful and failed cases. Arrows show the loadings of original variables (diameter, depth, and height) on the principal components.

The Analysis of standardized cavity measurements revealed substantial multicollinearity between cavity depth and entrance diameter (r = 0.80; variance inflation factors 4.67–23.98), whereas nest height was largely independent of other traits (r < 0.10; Table 3). To address this, we performed a principal component analysis (PCA) on the three variables. The first two components accounted for 93.3% of the total variance (PC1 = 60.6% and PC2 = 32.8%; Table 4). The loadings indicated that PC1 represented the overall cavity size axis, with positive contributions from cavity depth and entrance diameter, whereas PC2 captured the height axis, which was nearly orthogonal to size (Table 5, Figure 3). PC3 accounted for minimal residual variance (6.7%) and was excluded from further analysis.

**Figure 4:**
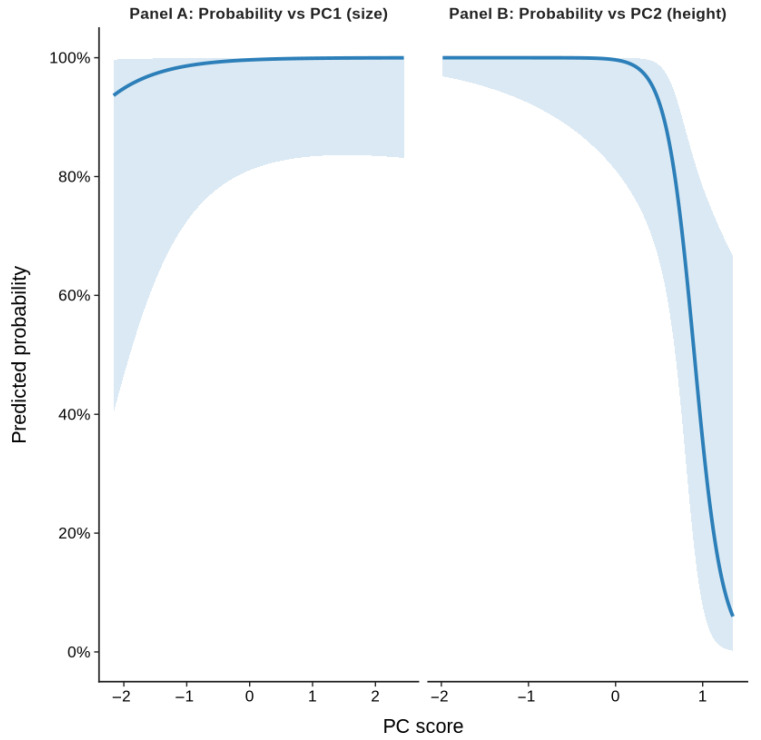
Predicted probability of reproductive success as a function of PCA-derived predictors. Solid lines indicate model-predicted probabilities from the fitted model, while shaded areas represent 95% confidence intervals around the predictions.

Logistic regression using PCA-derived components revealed a clear relationship between cavity structure and breeding success. PC2, which represents the height gradient (with lower PC2 values corresponding to taller cavities), showed a strongly significant correlation with success (Estimate = −6.215, OR = 0.002, 95% CI 1.1 × 10^−6^–0.095, *p* = 0.021). Because a higher cavity height is associated with lower PC2 scores, this negative coefficient indicates that taller cavities have substantially higher probabilities of success. PC1 (overall size axis) showed a weaker, marginally significant positive correlation (estimate = 1.355, OR = 3.87, 95% CI 1.21–41.62, *p* = 0.090; Table 6). The predicted probability curves and PCA biplot both support that nest height is the strongest predictor of breeding success, with a larger cavity size providing an additional but less pronounced benefit (Figure 3 and Figure 4).

A comparison of the models showed that PCA removed multicollinearity and resulted in orthogonal predictors that still had some explanatory power (AIC = 20.79, pseudo-R^2^ = 0.59, Table 4). GLM models suffered from separation and inflated variance when the raw predictors were used, and the estimates could be stabilized with Firth penalization, but at the cost of ease of interpretability. The PCA model enabled clear conclusions: entrance height is the primary driver of success, whereas the overall cavity size plays a secondary supporting role.

### 3.3. Breeding Success and Habitat Distances

**Figure 5:**
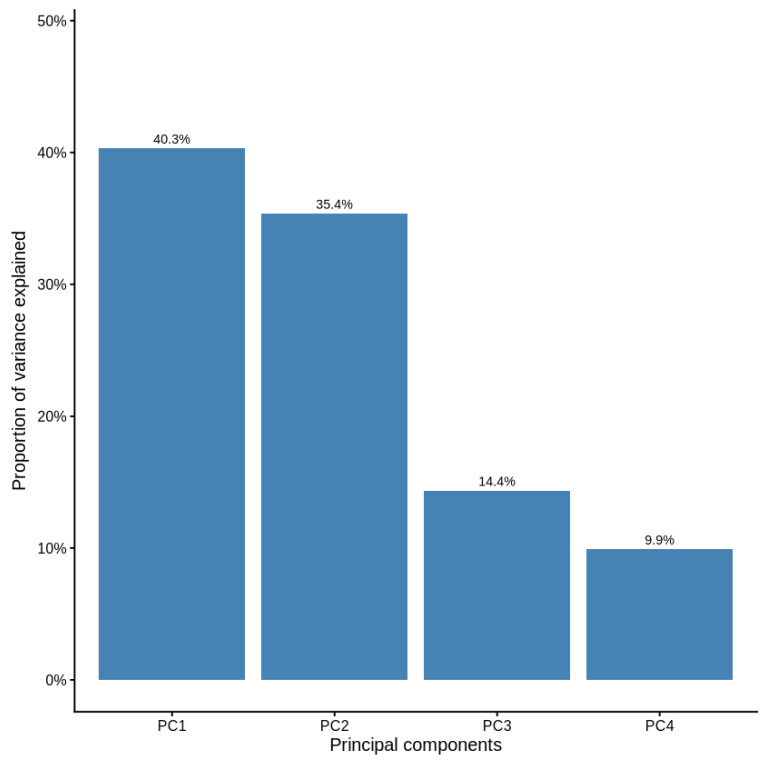
Scree plot showing the proportion of variance explained by each component.

**Figure 6:**
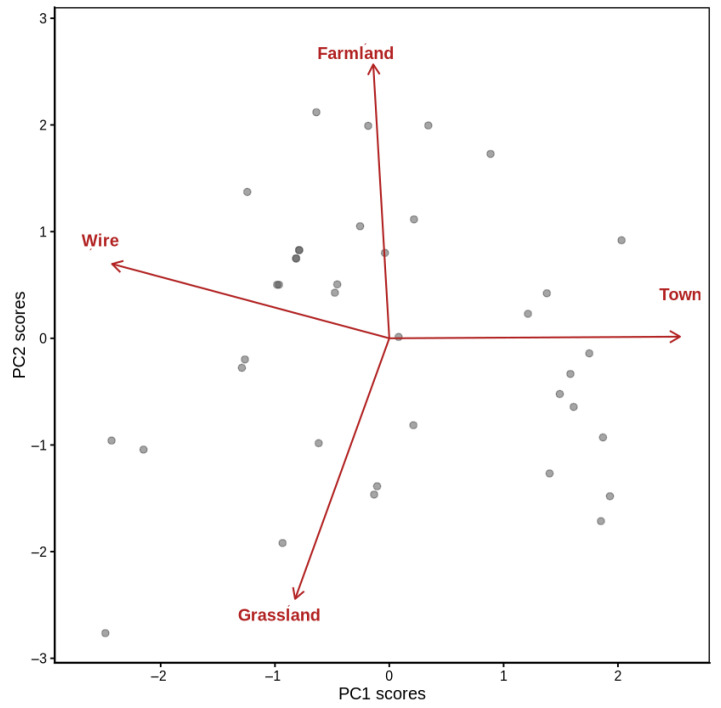
Principal component analysis (PCA) of the standardized habitat distances.

**Figure 7:**
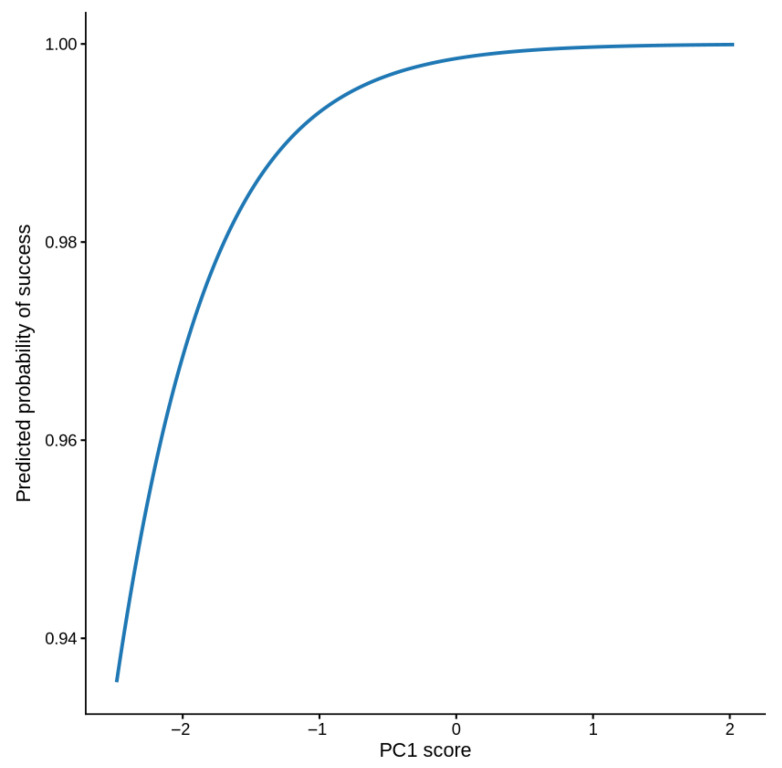
Predicted probability plot showing the effect of one principal component (typically PC1) on the probability of breeding success, with the other PCs held constant (usually at zero).

High variance inflation factors of the original distance variables were observed (VIF/original distances: 7.7–16.1), indicating severe multicollinearity. To address this issue, distance predictors were reduced using Principal Component Analysis (PCA). The first three principal components explained 90.1% of the total variation (PC1 = 40.3%, PC2 = 35.4%, and PC3 = 14.4%; Figure 5). PCA loadings revealed interpretable spatial gradients (Figure 6). PC1 represented the “town distance versus wire proximity” dimension, with higher PC values for sites farther from towns and closer to the electric wires. PC2 depicted a “grassland versus farmland” gradient, with low values for sites near grasslands and high values for sites far from farmland. Overall, PC3 indicated general remoteness from both grassland and farmland areas. Logistic regression using the three uncorrelated PCs showed that breeding success increased with PC1 and PC2, but decreased with PC3 (Table 7). Specifically, success was higher at sites farther from towns and closer to wires (PC1: β = 1.55, *p* = 0.10) and where grasslands were nearby but farmland was distant (PC2: β = 5.48, *p* = 0.09). Success declined at sites remote from both habitats (PC3: β = −2.75, *p* = 0.09). The model fit was strong (AIC = 18.0; Figure 7). Overall, PCA removed multicollinearity and uncovered consistent ecological gradients correlated with nesting success: proximity to grassland and moderate anthropogenic features (electric wires) favored successful breeding, whereas isolation from productive habitats reduced success. However, none of the individual habitat predictors were statistically significant.

### 3.4. Reproductive Biology

**Figure 8:**
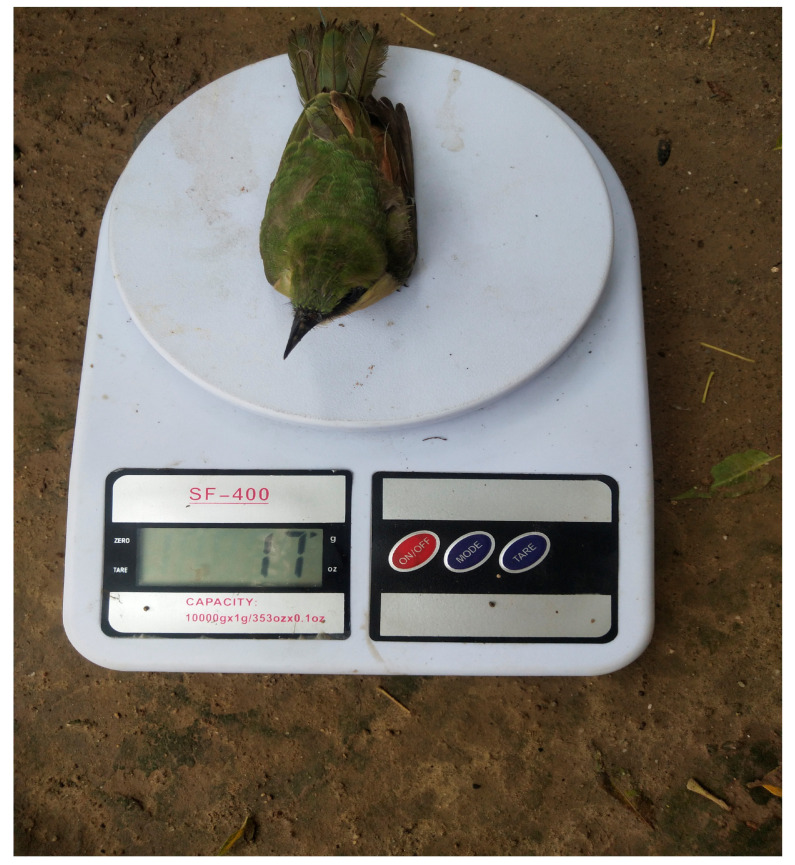
A well-grown chick of a small bee-eater.

Small bee-eater eggs were round, smooth, and white, and were laid in sequence within nest burrows from early March to late May. We measured 38 nests with 108 eggs, and these had the following average ± SD: weight 3.15 ± 0.12 g (range 2–4 g), width 16.26 ± 0.32 mm (13–19 mm), length 21.84 ± 0.6 mm (21–22 mm), and volume 2.98 ± 0.11 cm^3^ 1.98–4.05 cm^3^) (Figure S1). Egg-laying dates spanned from March to late May (Figure S2). Nests were typically excavated at an average height of 201.00 ± 13.87 cm. The average clutch size was 3.89 ± 0.82 eggs (range: 3–5), and the average brood size was 2.97 ± 0.9 nestlings (range: 1–5). Incubation began only after the final egg was laid and was shared by both parents, lasting an average of 15.52 ± 0.71 days (15–17 d). The brood-rearing period averaged 25.02 ± 0.87 days (24–26), resulting in 1.80 ± 0.13 fledglings per nest (1–3) (Figure 8 and Figure S3). Across all nests, 148 eggs were laid, of which 35 (1.40 ± 0.10 per nest) did not hatch. Of the 113 hatched nestlings, 46 (1.76 ± 0.20) were lost, and 11 (1.22 ± 0.14) died during the brooding period. Predators included snakes and Indian grey mongooses, the latter of which were directly observed under nest sites. The Jugger falcon was also seen attacking nest tunnels, although no direct predation was recorded. The fledging period extended from April 23 to July 7 (Figure S4). Overall, the hatching success rate was 77.5 ± 3.3%, fledging success was 51.2 ± 5.4%, and total breeding success—defined as the proportion of laid eggs resulting in fledglings—was 37.1 ± 3.8% (Table S1).

## 4. Discussion

This study provides quantitative data showing that nest structural components, particularly cavity depth and nest height, are positively associated with reproductive success in small bee-eaters. Cavity depth correlated with clutch size but not with fledging success, and nests at greater heights tended to produce more chicks. Meanwhile, the surrounding landscape attributes had either weak or non-significant correlations, which indicates that nest-site characteristics were more closely correlated with breeding success than habitat context.

### 4.1. Observations on Reproductive Biology

This study provides the first quantitative assessment of the correlation between nest architecture and the breeding ecology of small bee-eaters in Pakistan. Breeding occurs from March to July. Refs. [12,13] reported a breeding season from April to June, and other studies found February–August as the breeding season, with a peak in April–May in Bangalore. Our results suggest that the onset of breeding may coincide with periods of higher food availability, as reported in previous studies of the species [12], although prey abundance was not directly measured in the present study [15].

All detected nests were on sandy banks of Ballar stream, which is in accordance with an earlier finding that bee-eaters prefer to construct nest burrows on sandy riverbanks [16,17]. The same substrate preferences were observed in other soil-excavating species, the white-throated kingfisher (*Halcyon smyrnensis*) [18], and sand martin or bank swallow (*Riparia riparia*) [19], which also construct nests in sandy soils. Sandy soils, which have weaker cohesion, density, and moisture than clay soils, may promote excavation and improve nest ventilation [20,21]. Bee-eaters not only prefer sandy soils but also appear to excavate long tunnels in these substrates. Presumably, this behavior provides them with a microclimate advantage or helps them avoid predators.

The cavity depths in the present study were 65–120 cm, which is close to field observations of 100–200 m horizontal cavities in riverbanks and sandy soils [22]. In this study, nesting sites were located near mosaic farmlands, grasslands, and electric wires. These places offered many places to perch, such as small trees, branches, and electric infrastructure, to watch over nests and hunt [12]. The surrounding agricultural land also offers an abundance of protein-rich insect prey for both adults and nestlings. Notably, the small bee-eaters did not choose to nest in dense forest areas. They preferred to nest on open riverbanks with sparse vegetation. This choice is consistent with what has been observed in previous studies on nesting patterns across species of bee-eaters [20].

The reproductive success of the small bee-eater is also susceptible to predation, especially in areas with reduced vegetation cover, where nest exposure may have increased. Although several potential predators (e.g., snakes, Indian grey mongooses, and house crows) were identified in the study area, no direct predation events were observed at the nests. This absence of direct evidence may reflect the species’ use of deep cavities that limit visual detection of predation, infrequent timing of nest checks, or nocturnal activity patterns of predators [23,24]. Future work using camera monitoring could provide clearer documentation of predation events and predator identity. In this study, egg measurements averaged 21.84 ± 0.6 mm in length, 16.26 ± 0.32 mm in width, and 3.15 ± 0.12 g in weight. Asokan (1995) [13] reported similar dimensions, with egg lengths of 21.0 mm, widths of 18.0 mm, and weights of 2.62 g. This agrees with earlier reports that indicated clutch sizes averaging 2–7 eggs [25], and is in line with observations in other species of bee-eaters, such as the black-headed bee-eater (*Merops breweri*) [26], white-fronted bee-eaters (*M. bullockoides*), and European bee-eaters (*M. apiaster*) [27]. The likely influences of variation in clutch size include the age of the female and the availability of resources during the nesting period. The observed breeding success (37.1%) is slightly lower than the values reported for small bee-eater breeding in southern India (≈79.13%) [13], but similar to those of other species of cavity-nesting birds, such as kingfishers and sand martins, suggesting that the reproductive performance is moderate under natural conditions [12]. This suggests that the number of eggs laid by the small bee-eater species in the current study was offset by lower fledging rates, which reduced the species’ overall reproductive success. It is important to note that these findings are based on observations from a single breeding season. Inter-annual variations in rainfall, temperature, and predator abundance could substantially influence breeding success in subsequent years, as has been reported for other cavity-nesting and insectivorous bird species [28]. Therefore, long-term monitoring across multiple seasons would be valuable for determining whether the breeding success recorded here remains consistent under varying environmental conditions [29,30].

### 4.2. Effect of Nest Dimension and Habitat Features on Reproduction

Our results show that cavity depth and nest height are significantly correlated with reproductive outcomes in small bee-eaters, partly in line with foundational and contemporary literature [4,6,26,27,29,30,31,32,33,34,35]. Ref. [32] showed that fine-scale cavity morphology, including the nesting cavity entrance/depth ratio, was primarily adaptive in terms of excluding native predators, rather than directly determining clutch size or fledging success. Instead, they found that cavity size does not directly affect reproductive success but indirectly affects it through predator access [32]. Previous studies have found significant correlations between nest space and clutch size under experimental conditions. Our results were derived from natural cavities and correlated. Previous studies have indicated that larger cavity dimensions are related to higher reproductive investment [4,33]. However, the underlying reasons for this are an ongoing topic of debate. For example, the direct causal relationship revealed by [34,35] between nest space and clutch size was established by manipulating the floor area of nest boxes. These studies found that birds adjusted their reproductive output depending on perceived spatial constraints. Along the same lines, ref. [6] showed that not only the cup but also the overall nest structure serves as cues for reproductive allocation.

However, our study differs in several important ways. First, it studies field conditions using naturally excavated nests, avoiding artificial nest box constraints that could bias behavior [4]. Second, while many studies focus on the base area of the nest, our analysis shows that the vertical dimension of the nest plays an especially strong role in determining reproductive outcomes. This may suggest that cavity length, rather than just floor area, is a more relevant cue in species such as bee-eaters. This is in contrast to temperate cavity-nesters [34,35]. The small bee-eater breeds in a warmer, more open habitat, where nest depth could be more tightly linked to thermal buffering and moisture retention, which are indirect benefits that may influence female reproductive decisions. These results offer further support for the clutch-size phenotype plasticity hypothesis regarding nest structures [4,6]. Our finding that cavity depth was strongly correlated with clutch size (CMP: β = 0.46, *p* = 0.036) adds to the evidence that cavity quality limits or facilitates reproductive investment in excavating birds. However, these are potential explanations that warrant future testing rather than established processes. Two non-exclusive mechanisms are the most plausible explanations for this pattern:

First, microclimatic buffering inside deeper cavities may reduce thermal stress on eggs and incubating adults, lowering energy demands and thereby allowing greater reproductive investment (larger clutches) [36,37]. Experimental and observational studies across taxa have shown that nest architecture (depth, cavity volume, and insulation) can alter within-nest temperatures and cooling rates, with consequences for parental workload, incubation efficiency, and clutch and brood size [38]. These thermal pathways are realistic for bee-eaters that nest in exposed earthen banks, as deeper burrows likely moderate diurnal temperature extremes [4]. Future experimental or multi-year studies would be valuable to test whether microclimatic stability, predator avoidance, or other factors directly drive these observed relationships.

Second, deeper cavities can reduce predation or parasitism risk by making access more difficult for mammals, snakes, or opportunistic avian predators, and by reducing detection probability [39]. Theoretical and empirical studies on cavity nesters link safer cavity features to higher reproductive investment; where predation risk is lower, parents can afford larger clutches because the probability of offspring survival to fledge increases [40]. This predator-mediated interpretation is consistent with general findings from cavity-nesting literature and with studies showing that cavity dimensions influence predation outcomes [40,41,42]. Because our data were observational, we could not partition thermal from predation mechanisms; both could operate together to make deeper cavities a higher-quality reproductive site [42].

Comparative studies on cavity-nesting and excavating species further support a depth–clutch relationship, although effect sizes and directions vary across species’ life histories and environmental contexts. For example, ref. [42] showed that clutch size evolution in excavators depends on trade-offs among nest site availability, excavating ability, and diet-mediated energy budgets, where deeper or larger cavities reduce risk or energetic costs, and larger clutches become feasible. Thus, our results fit a broader pattern in which cavity quality is a key axis of reproductive variation among and within species.

We found that the structural height of nest cavities had a strong correlation with breeding success, and that the total cavity size (cavity depth + entrance diameter) had an additional modest correlation. Using principal component analysis (PCA) to transform the highly correlated predictors into orthogonal axes, we resolved multicollinearity and obtained interpretable logistic regression estimates. Higher breeding success was associated with nests located higher above the ground, as reflected by lower PC2 scores (*p* = 0.021), whereas PC1 showed only a marginal positive association with success (*p* = 0.090). This pattern suggests that once the minimum cavity size is met, vertical placement of the nest becomes the most influential factor for reproductive success. This relationship is consistent with the idea that vertical placement can affect exposure to predator guilds as well as disturbance regimes; higher nests are often less accessible to many terrestrial predators and may experience less human disturbance, resulting in better nest survival and fledging success in some systems [43]. Recent empirical work shows that the presumed loss to predation frequently declines with increasing height once nests exceed a threshold (often several meters), although the patterns are species- and system-specific [43]. On our site, even modest height differences along earthen banks may change accessibility to small mammal or reptile predators and alter the frequency of disturbance by people or livestock, producing the observed patterns. However, correlations of nest height are often conditional; for example, avian predator risk can increase at higher elevations in some environments, and the benefits of elevation may only appear above a critical height [44]. Because our nests occur in earthen banks and height variation is limited compared with tree-nesting species, the detected positive correlation should be interpreted as a local correlational result rather than a universal rule. Experimental translocations or camera-documented predator activity would help test whether height mediates predation or disturbance at our site.

Cavity depth and entrance diameter are considered relevant nest structural characteristics that may be associated with adult comfort/nestling space and predator avoidance [45,46]. In our PCA, they showed high positive loadings on PC1 (size effect). The positive coefficient of PC1 (OR ≈ 3.9) indicates that larger cavities tend to increase the probability of success, but the wider confidence interval and marginally significant *p*-value indicate that beyond a certain point, increasing cavity size may have diminishing returns.

We found only weak or marginal correlations between the measured habitat distances (grassland, farmland, towns, and electric infrastructure) and fledging success. This pattern can be explained by several reasons. Bee-eaters are long-range aerial foragers, and foraging distances during the breeding period often exceed those in the immediate vicinity of the nesting sites. Hence, relatively coarse local-level distance metrics may not adequately reflect the actual resource landscape experienced by breeding individuals in their habitat. Moreover, the distances we quantified were direct proxies of landscape composition; they did not account for quantitative aspects of prey base (cavity abundance), pesticide exposure, and microhabitat heterogeneity, which often determine reproductive performance. Finally, the relatively small sample size and single-season focus minimized the power to detect subtle habitat relationships. This might encourage the detection of stronger structural correlates, such as cavity depth or nest height. Further studies that sample prey and incorporate finer-grain landscape metrics with multi-season replication would help disentangle this habitat-context effect.

### 4.3. Implications for Conservation

While our results are correlational, they indicate that the characteristics of the nest structure, in particular, cavity depth and nest height above the ground, are positively related to fledging success. Conservation of geomorphological features that allow natural cavity digging, such as stable earth banks, and reducing human disturbance around riverbanks would assist local breeding populations in these respects. These conservative measures should be strengthened through additional research on foraging habitat quality to better understand bee-eater breeding ecology and implement appropriate management strategies.

### 4.4. Study Limitations and Future Directions

This study was restricted to a single stream system, a small sample size, and one breeding season. Predation was only passively inferred, and the return of the observers to each nest at intervals could have been disturbing to the birds. Moreover, other ecological variables (such as prey availability and vegetation cover) were not quantified in this study. Such analyses should be replicated and extended with more extensive multi-seasonal sampling in various habitats. We recommend that (1) motion-activated cameras be used to detect nest predators and disturbance events, (2) cavity microclimate be measured to test the thermal-buffering hypothesis, (3) prey availability and pesticide exposure inside foraging areas be assessed, and (4) monitoring efforts continue across multiple years at additional sites to determine the consistency of patterns. The most direct test of causality involves experimentally offering artificial cavities at different depths [47].

## 5. Conclusions

This study is the first quantitative assessment of the breeding biology of small bee-eaters in Pakistan, illustrating that nest construction characteristics are correlated with reproductive success. Clutch size was strongly correlated with cavity depth, suggesting that deeper cavities may lead to a higher reproductive output through greater microclimatic stability or protection from predators. Similarly, breeding success was positively correlated with nest height, suggesting that higher nests might confer benefits for chick survival in the long term.

In contrast, larger-scale landscape features had weaker correlations with breeding success, and the characteristics of the nesting microhabitat seemed to be more important in shaping reproductive endpoints for this species. Conservation measures should thus concentrate on preserving suitable nesting sites (i.e., stable sandy/earthen banks) and preventing human disturbance to active colonies. Protecting the physical environment for natural cavity excavation can help maintain local breeding populations and assist in the long-term conservation of these species. Because our results were derived from one breeding season at a single site, multi-year and multi-site studies are needed to confirm whether these patterns hold more broadly.

## Figures and Tables

**Table 1:** Morphometric characteristics of bee-eater nests.

Variable	Mean ± SD	Range (Min–Max)	*N*
Cavity depth (cm)	86.68 ± 18.30	65–120	38
Entrance diameter (cm)	10.89 ± 2.09	7–14	38
Nest height (cm)	201.00 ± 85.47	88–380	38

**Table 2:** CMP Regression model testing the relationship between nest characteristics and clutch size.

Term	Estimate (β)	Std. Error	z Value	*p* Value
(Intercept)	44.6267	55.5492	0.803	0.422
Depth (cm)	0.4568	0.2178	2.097	0.036
Diameter (cm)	2.5032	1.5604	1.604	0.109
Height (cm)	0.0101	0.0228	0.442	0.659

**Table 3:** Correlation matrix of standardized predictors.

Variable	depth_cs	diameter_cs	height_cs
Depth	1.000	0.800	−0.086
Diameter	0.800	1.000	−0.081
Height	−0.086	−0.081	1.000

A high correlation between depth and diameter (r = 0.80) indicated multicollinearity, whereas other correlations were weak.

**Table 4:** The eigenvalues and variances are explained by the principal components.

PC	Eigenvalue	Proportion of Variance	Cumulative Proportion
PC1	1.8169	0.6056	0.6056
PC2	0.9830	0.3277	0.9333
PC3	0.2001	0.0667	1.0000

**Table 5:** PCA loadings (rotation matrix) for the standardized predictors.

Variable	PC1	PC2	PC3
Depth	0.7001	−0.0974	0.7074
Diameter	0.6996	−0.1047	−0.7068
Height	−0.1429	−0.9897	0.0051

**Table 6:** Logistic regression using PCA-derived predictors (PC1 and PC2).

Term	Estimate	Std.	z-Value	*p*-Value	OR	CI Lower	Cl Upper
Intercept	5.621	2.126	2.64	0.008	276.20	12.11	87,754.94
PC1 (Size)	1.355	0.798	1.70	0.090	3.87	1.21	41.62
PC2 (Height)	−6.215	2.702	−2.30	0.021	0.002	1.13 × 10^−6^	0.095

PC2, which mainly represents height variation, was retained as a significant predictor of success (*p* = 0.021), again, stressing the importance of height in shaping the success. PC1 (size) showed a positive trend without significance (*p* = 0.090). The PCA logistic model had an AIC of 20.79 and a McFadden pseudo-R^2^ of 0.59, showing good explanatory capacity and less multicollinearity.

**Table 7:** Results of the logistic regression analysis with PCA components as breeding-success predictors. Breeding success (1 = success, 0 = failure) was the response variable, and the first three principal components (PC1–PC3) were the predictors. The model was fitted using a logit link. AIC = 18.0.

Predictor	Estimate (β)	Std. Error	z Value	Pr (>|z|)
Intercept	6.5200	3.6557	1.783	0.0745
PC1	1.5480	0.9505	1.629	0.1034
PC2	5.4781	3.2222	1.700	0.0891
PC3	−2.7467	1.6050	−1.711	0.0870

## Data Availability

All primary nest measurement and habitat distance data used in this study are publicly available on Figshare (DOI: https://doi.org/10.6084/m9.figshare.30630062).

## Supplementary Materials

The following supporting information can be downloaded at https://www.mdpi.com/article/10.3390/ani16020186/s1, The additional file contains one table (Table S1) and four figures (Figures S1–S4) that present specific breeding success measures and egg morphometrics data that underpin the results of this study. Table S1. Overall breeding success in small bee-eaters. Figure S1. Egg weight, egg length, egg width and egg volume of bee-eater eggs. Figure S2. The egg-laying dates of small bee-eaters. Figure S3. The violin box plot shows the average breeding parameters and nest height of small bee-eaters. Figure S4. The number of young fledged throughout the breeding season in small bee-eaters.
