# Supplementary material for: Nest Depth and Height Are Associated with Breeding Outcomes in the Small Bee-Eater (Merops orientalis): A Preliminary Field Study from Pakistan
Source: Animals (Basel). 2026 Jan 8;16(2):186. doi: 10.3390/ani16020186 (PMC12837328; doi:10.3390/ani16020186)
Supplement: Supplementary file 1 [file animals-16-00186-s001.zip › animals-3975467-supplementary.pdf]

Supplementary Information

Nest Depth and Height Are Associated with Breeding Outcomes in the Small Bee-Eater (*Merops orientalis*): A Preliminary Field Study from Pakistan

Asif Sadam [1], Muhammad Awais [2], Huijian Hu [1], Dongmei Yu [1] and Yiming Hu [1]

[1] Guangdong Key Laboratory of Animal Conservation and Resource Utilization, Institute of Zoology, Guangdong Academy of Sciences, Guangzhou 510260, China
[2] Department of Zoology, Islamia College, Peshawar 25120, Pakistan

Email address:

AS, saddamasif2@gmail.com

MA, mawaisicp@gmail.com

YH, huyiming@giz.gd.cn

YD, yudongmei50@163.com

HH, 13570909977@139.com

* **Corresponding author:** Huijian Hu (13570909977@139.com)

The following supplementary table and figures support the main manuscript.

Table S1. Overall breeding success in small bee-eaters.

Variable	Range	Mean ± SE
Hatching success	25-100	77.5 ± 3.3
Fledging success	0.0-100	51.2 ± 5.4
Breeding success	0.0-75	37.1 ± 3.8

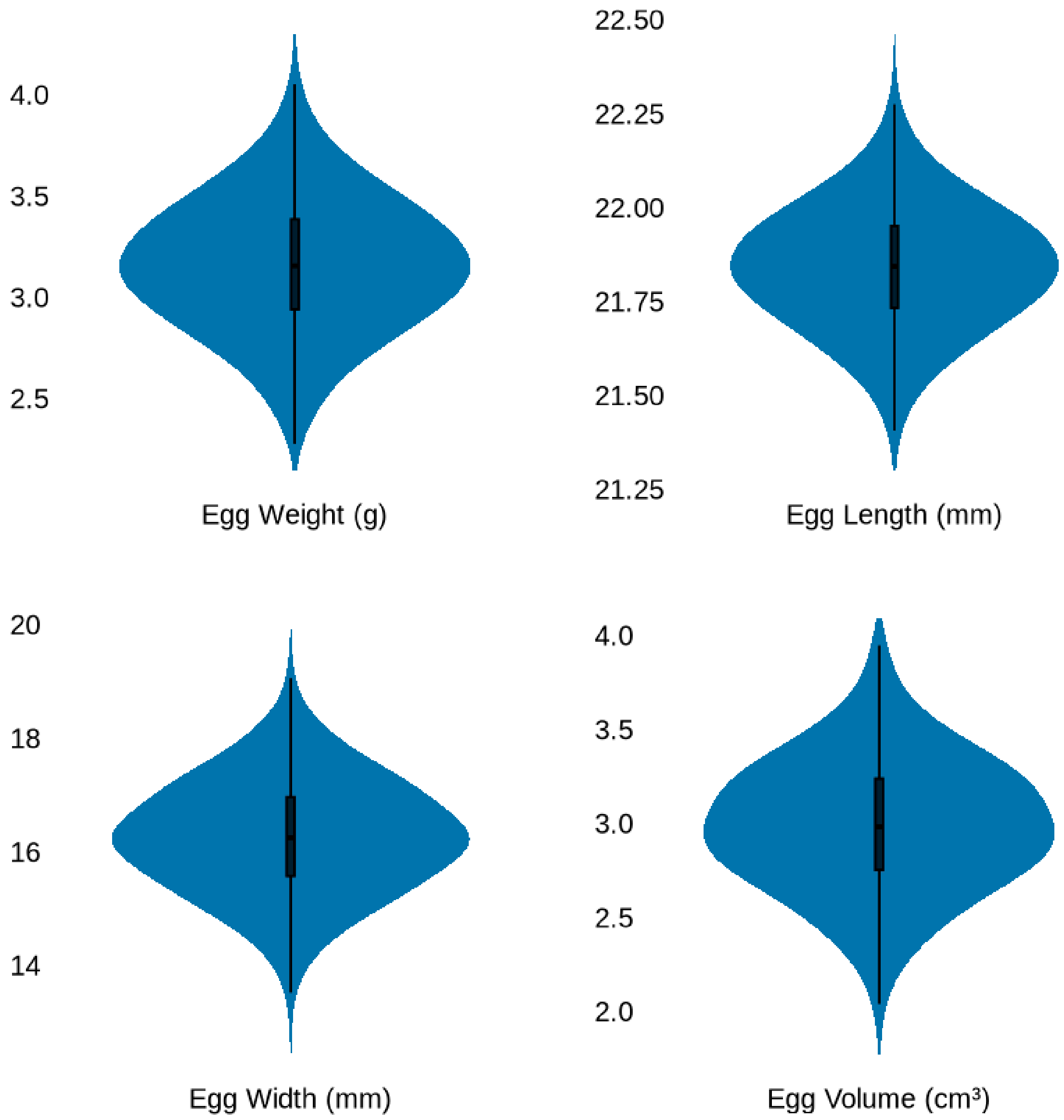

Figure S1. Egg weight, egg length, egg width and egg volume of bee-eater eggs.

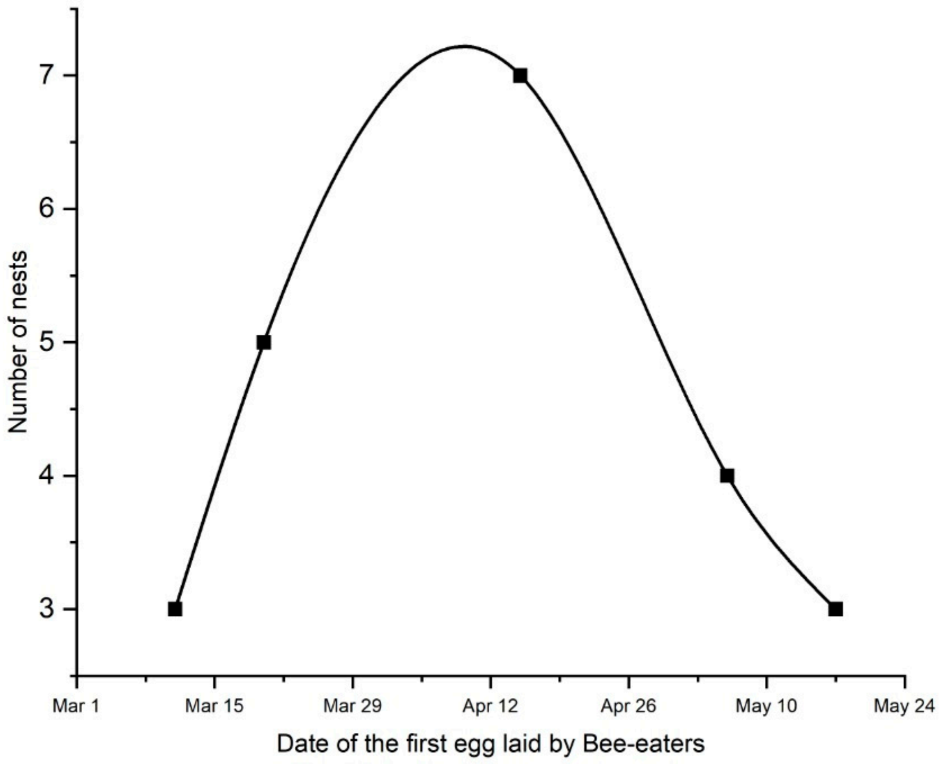

Figure S2. The egg-laying dates of small bee-eaters.

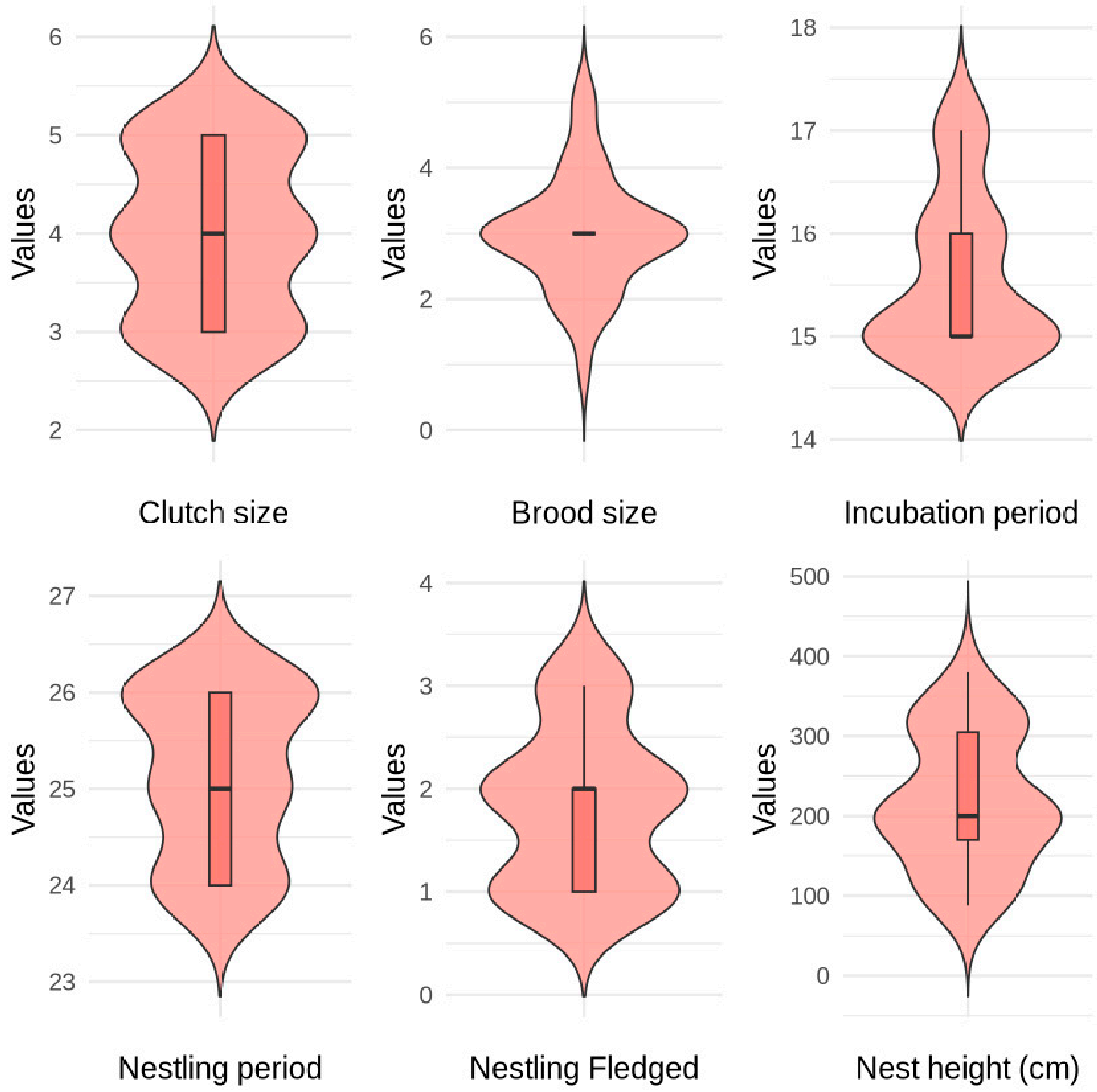

Figure S3. The violin box plot shows the average breeding parameters and nest height of small bee-eaters.

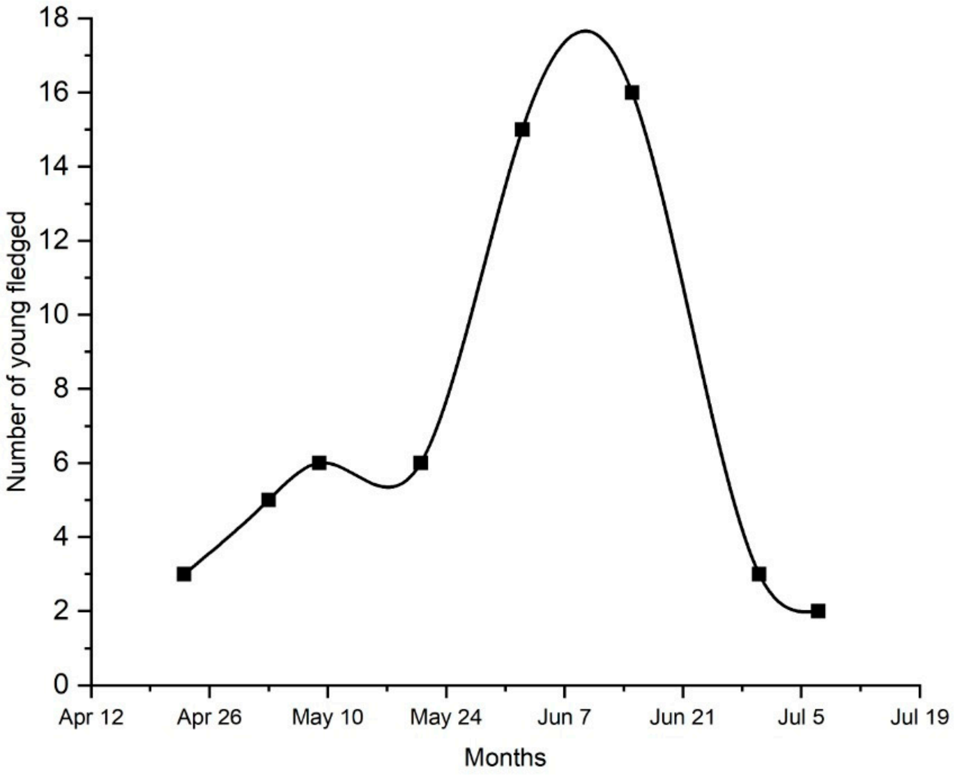

Figure S4. The number of young fledged throughout the breeding season in small bee-eaters.
